# Additive Manufacturing of Metallic and Ceramic Components by the Material Extrusion of Highly-Filled Polymers: A Review and Future Perspectives

**DOI:** 10.3390/ma11050840

**Published:** 2018-05-18

**Authors:** Joamin Gonzalez-Gutierrez, Santiago Cano, Stephan Schuschnigg, Christian Kukla, Janak Sapkota, Clemens Holzer

**Affiliations:** 1 Polymer Processing, Department of Polymer Engineering and Science, Montanuniversitaet Leoben, Otto Gloeckel-Strasse 2, 8700 Leoben, Austria; santiago.cano-cano@unileoben.ac.at (S.C.); stephan.schuschnigg@unileoben.ac.at (S.S.); clemens.holzer@unileoben.ac.at (C.H.); 2 Industrial Liaison Department, Montanuniversitaet Leoben, Peter Tunner Strasse 27, 8700 Leoben, Austria; christian.kukla@unileoben.ac.at

**Keywords:** additive manufacturing, fused filament fabrication, material extrusion, 3D-printing, highly-filled polymers, metals and ceramics

## Abstract

Additive manufacturing (AM) is the fabrication of real three-dimensional objects from metals, ceramics, or plastics by adding material, usually as layers. There are several variants of AM; among them material extrusion (ME) is one of the most versatile and widely used. In MEAM, molten or viscous materials are pushed through an orifice and are selectively deposited as strands to form stacked layers and subsequently a three-dimensional object. The commonly used materials for MEAM are thermoplastic polymers and particulate composites; however, recently innovative formulations of highly-filled polymers (HP) with metals or ceramics have also been made available. MEAM with HP is an indirect process, which uses sacrificial polymeric binders to shape metallic and ceramic components. After removing the binder, the powder particles are fused together in a conventional sintering step. In this review the different types of MEAM techniques and relevant industrial approaches for the fabrication of metallic and ceramic components are described. The composition of certain HP binder systems and powders are presented; the methods of compounding and filament making HP are explained; the stages of shaping, debinding, and sintering are discussed; and finally a comparison of the parts produced via MEAM-HP with those produced via other manufacturing techniques is presented.

## 1. Introduction

Additive manufacturing (AM) is a technology for fabricating real three-dimensional (3D) objects, using metals, ceramics, or plastics, which may be used in various applications [1]. AM is defined by International Organization for Standardization (ISO) and American Society for Testing and Materials (ASTM) as the “process of joining materials to make parts from 3D model data, usually layer upon layer, as opposed to subtractive and formative manufacturing methodologies”. The processes encompassed in AM are the 3D analog of the very common 2D digital printers; therefore, AM is also commonly referred as 3D printing. However, in the last 30 years, AM has also been referred to as direct digital manufacturing, additive layer manufacturing, additive fabrication, additive techniques, additive processes, free-formed fabrication, solid free-formed fabrication, rapid manufacturing, and rapid prototyping [2]. The term additive manufacturing has been accepted by the ASTM F42 Technical Committee and the ISO Technical Committee TC261 and this has contributed to the international adoption of this term [3]. There are several variants of AM processes available today, but the whole process can be summarized as follows [1,3]:

1. Design concepts are generated from scratch or using 3D scanners, computed tomography (CT) scans, or magnetic resonance imaging (MRI) in the case of medical implants.
2. A 3D computer aided design (CAD) model is prepared.
3. The CAD model is analyzed and optimized with the aid of computer optimization techniques such as finite element analysis (FEA).
4. The CAD model is commonly transformed into a Standard Triangulation or Tessellation Language (STL) file and imported into an AM setup. Nevertheless, the STL format lacks many features such as color or materials in the parts. For these reasons a new format was implemented by ASTM ISO, the Additive Manufacturing File Format (AMF) [4]. Besides AMF, more than 30 other alternatives to the STL file exist, three important examples of which are OBJ, PLY, and 3MF [5]. This last one (3MF) is supported among others by Microsoft, Autodesk, Dessault Systemes, 3D Systems, Materialise, Ultimaker, Mcor, PTC, FIT, GE, EOS, HP, Siemens PLM, nTopology, SLM solutions, Stratasys, and Shapeways [6]. Only time will tell which file format becomes the standard file in the future.
5. The geometric shape in the STL or other format files is sliced into thin layers and the movement of the depositing or fusing unit (“printing head”), and substrate (“printing platform”), as well as other parameters are programmed by specialized software that prepares the G-code, which is a numerical control programming language.
6. The AM machine builds the tridimensional object layer by layer with the specified parameters.
7. The built part is removed from the building platform and the removal of support structures used to build complex geometries is conducted. The excess unbound building material needs to be removed in a cleaning step depending on the type of AM technique used.
8. After the object is removed and cleaned, it might require further post-processing such as polishing, coating, or thermal treatment to obtain a functional part.

The main advantage of AM over conventional manufacturing processes is dealing with geometric and material complexities that cannot be created, technically and/or economically, using subtractive and formative manufacturing processes [7]. AM has the possibility to create structures that can be very light, stable, and at the same time contain features with a high degree of functionality [2]. The cost of producing a part using AM techniques is almost independent of the number of parts that are needed to be produced since there are no tooling costs associated with the process [3]; thus, AM is ideal for unique parts that are manufactured in low production volumes. For this reason, AM has strong usage in medical and dental applications [8,9,10]. AM allows the simulation of implant designs prior to their manufacturing and allows for customization for each individual patient. Thus, AM helps to reduce the costs and time required to manufacture fitting implants [1]. However, the medical field is not the only one that benefits from the use of AM; AM has many significant applications in the automotive, aerospace, and energy fields [7]. This is reflected in a drastic increase of AM fabricators, the number of parts produced, and materials used in AM between 2010 and 2015, with an annual growth rate of approximately 30% [3]. The constant evolution of production and design techniques using AM will make the technology even more cost-effective and efficient in the future. As such, the use of AM with industrial metals and ceramics will continue to grow.

Over the last three decades, many AM technologies have been developed. The standard EN ISO/ASTM 52921:2017 [11] defines the different AM technologies as shown in Table 1. This table also gives alternative names, the materials that are processable, and the strengths and weaknesses of each technique. Alternative names for the different types of additive manufacturing include: 3D printing (3DP) [1], selective laser sintering (SLS) [12], laser engineered net shaping (LENS) [12], selective laser melting (SLM) [13], direct laser metal fabrication (DLMF) [14], electron beam melting (EBM) [15], stereolithography (SLA) [16], high speed sintering (HSS) [17,18], laminated object manufacturing (LOM) [19], and fused deposition modeling (FDM) [20], also known as fused filament fabrication (FFF) [21]. Details of each of these processes have been described in the cited references [1,2,3,7,8,9,10,11,12,13,14,15,16,19,20,21,22,23]. As it can be seen, the different techniques can be used for different applications and with different materials. Thus, it can be said that one technique complements another.

The focus of this review is on material extrusion additive manufacturing with highly-filled polymers (MEAM-HP) with a particular emphasis on its application for the fabrication of metallic and ceramic components. MEAM-HP, in this case, is a multi-step/indirect process, which makes use of a sacrificial polymeric binder material to shape metallic and ceramic powder particles. The polymeric binder is usually removed in subsequent (catalytic, solvent, and/or thermal debinding) treatments and the powder particles are bonded together in a conventional sintering step.

This review paper is organized in six subsequent sections following this introduction. Section 2 explains the different types of MEAM currently available. Section 3 introduces the process of MEAM-HP and the materials used for the production of metal, ceramics, or metal-ceramic components. Section 4 describes the procedure of building parts with MEAM. Section 5 describes the post-shaping operations needed to obtain metal, ceramics, or metal-ceramic components. Section 6 offers a comparison between MEAM-HP and other processing technologies used to produce similar parts. Finally, Section 7 offers a summary of the review paper and perspectives for further improving MEAM-HP.

## 2. Material Extrusion Additive Manufacturing (MEAM)

Material extrusion additive manufacturing (MEAM) consists of softening a material and pushing it through an orifice in order to deposit that material in layers to build a 3D structure [23]. Extrusion-based additive manufacturing processes are among the most widely used AM processes, particularly when working with polymers and thermoplastic composites [24]. Compared to other AM processes, the equipment used for MEAM can be inexpensive and very easy to operate [2,25,26]. Therefore, the main advantage of MEAM is the rapid or cheap reproduction of standard components or prototypes with a variety of polymeric materials, even with low melting temperature metallic alloys [2,27].

Unlike other AM techniques, extrusion-based additive manufacturing techniques are well suited for multi-material deposition and can be used for a wide range of thermoplastic materials [2,3,22,23,26]. In general, most of the MEAM machines are equipped with a single extrusion head, but there is the possibility of adding two or more extrusion units to allow for multi-material fabrication [2,24]. Meanwhile, the growing interest in additive manufacturing is focusing currently to create high value of the technology by developing and validating new materials and novel applications of fabricated parts.

### 2.1. Types of Material Extrusion Additive Manufacturing

**Figure 1:**
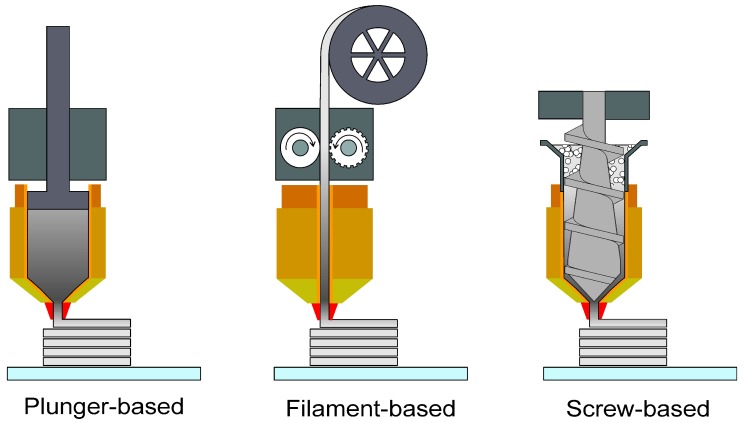
Different types and approaches for extrusion-based additive manufacturing.

The basic principle of material extrusion additive technology involves the loading and liquefaction of the material, moving the material through a nozzle or orifice by applying force or pressure, plotting liquefied material according to a pre-defined path in a controlled manner, and layer-by-layer bonding of the material to itself or a secondary build material to form a coherent solid structure [2]. After a layer is completed, the build platform moves down or the extrusion head moves up, and a new layer of material is deposited and adhered onto the previous layer. Whenever necessary, support structures are included in the process to enable the fabrication of complex geometrical features. This basic principle enables the production of complex parts without a shaping tool other than a die with a simple geometry, generally round. Depending on the type of extruder used, one can classify material extrusion additive manufacturing into different types [28], which will be described in the following section and schematically shown in Figure 1.

#### 2.1.1. Material Extrusion with Plungers

Two companies based in the USA, Desktop Metal Inc. and Markforged Inc. [29,30], currently offer MEAM machines that use special profiles (rods) made up of metal or ceramic powder with a thermoplastic binder system. Desktop Metal calls their process bound metal deposition^TM^ and Markforged calls theirs atomic diffusion additive manufacturing (ADAM). The profiles are fit into cartridges and are then fed into a plasticizing unit where the highly-filled thermoplastic composite is soft enough for extrusion. The soft material accumulates in a reservoir and finally a mechanical drive system (e.g., plunger) pushes the soft material and deposits it onto the building platform in a layer-by-layer manner [29,30,31]. It can be seen that these machines are very similar to the machines used in robocasting [32,33], with the exception that the building materials have a thermoplastic material as a binder, while in robocasting water is used as a binder. Another particular difference is that the machine patented by Desktop Metal Inc. has an ultrasonic vibrator with sufficient energy to ultrasonically bond an extruded building material onto the previously deposited layers [29]. Alternatively, the machine by Markforged Inc. has a laser scanning displacement sensor on the printed head that acts as an in-process inspection tool to ensure that the correct dimensions are being printed [30,31].

In general, ram extrusion machines with cartridges are meant to be used for shaping parts that eventually will be made out of only metal or ceramic, thus the rods have a large amount of powder and the printed parts are sintered to obtain a dense part. The rods use similar materials as used in the well-established process of powder injection molding (PIM) [34]. On their website, Markforged Inc. offers their proprietary binder system with powder of stainless steel (316L and 17-4PH), and advertises in-development face feedstocks with Inconel (625), titanium alloy (Ti-6Al-4V), tool steel (A-2 and D-2), and aluminum (6061 and 7075) [30]. The Markforged Inc. binder is thermally debound before sintering [35]. Desktop Metal Inc. advertises the development of feedstock materials with powders of stainless steel, high-performance steel, copper, tool steel, carbide, aluminum, heavy alloys, titanium, magnetics, low expansion metals, and superalloys [36]. The binder used by Desktop Metal Inc. is solvent debound before thermal debinding and sintering is done [36].

#### 2.1.2. Material Extrusion with Filaments

Material extrusion of filaments was first patented by the company Stratasys [2,37] and commercialized as fused deposition modeling or FDM^TM^. However, such a name could be applied to other AM techniques that melt materials and deposit them onto a platform or onto previously deposited layers of material, such as pneumatic extrusion, microinjection molding of droplets (e.g., Freeformer [38]), screw extrusion of pellets, and ram extrusion with rods. Therefore, an alternative terminology was introduced as fused filament fabrication or FFF [39]. Fused filament fabrication (FFF) is the most widely used MEAM technique. The main reasons for its popularity are its safe and simple fabrication process (i.e., no powders, lasers, solvents, nor volatile compounds are needed), the low cost of the equipment, and the availability of a great variety of filaments for printing. In the FFF process, the filament is extruded through a nozzle and deposited on a building platform one layer at a time, where it solidifies. When a heated chamber and/or heated building platform are available, the printing chamber and platform are kept at temperatures below the material’s melting point, but higher than room temperature to promote adhesion to the printed bed and to reduce thermally induced stresses [2,40,41]. Please note that even if a heated building platform and/or chamber are not available, it is still possible to perform MEAM with certain materials at room temperature [42].

FFF machines are ram extruders, with the filament being the ram that pushes the softened material out of the printing head. In conventional FFF machines the filament is first pulled by the driving wheels and then it is pushed by the same wheels into a liquefier and later into a nozzle. Therefore, sufficient mechanical strength is required for the filament to retain its shape after being forced through the drive wheels [43] to transfer the force into the liquefier. This transfer of force can be altered by a number of factors. First, the motors must generate sufficient torque. Next, the wheels must have enough friction with the filament to transfer the force from the wheels to the filament. At the same time, the filament must be strong enough to avoid shearing due to the pinching from the wheels. Finally, the filament must not buckle between the drive wheels and the entrance to the liquefier. That is, the force transferred from the drive wheels to the filament should be efficiently transferred into the center of the liquefier in the direction of the melt flow, with minimal loss due to filament buckling and compression [2,22,43]. In addition to these requirements, the filament should also be flexible enough to be spooled, so that the filament can be easily stored in a compact place and fed in a continuous manner into the liquefier [15,43]. As it can be expected, not all materials can fulfill all of these conditions, yet numerous thermoplastics-based materials are available as filaments for FFF.

The most common non-filled thermoplastic materials used in FFF are acrylonitrile butadiene styrene (ABS) and polylactic acid (PLA). However, other examples of non-filled thermoplastics filaments commercially available include: acrylonitrile styrene acrylate (ASA), polyamide (PA), polycarbonate (PC), polyphenylsulfone (PPSF, PPS, or PPSU), polyetherimide (PEI), thermoplastic polyurethane (TPU), polyethylene terephthalate (PET), thermoplastic elastomer (TPE), high impact polystyrene (HIPS), polyvinyl alcohol (PVA), polyether ether ketone (PEEK), polyvinylidene fluoride (PVDF), polyoxymethylene (POM), polyhydroxyalkanoate (PHA) blended with PLA, and some other blends of the previously mentioned polymers [1,24,26,44]. Examples of composite materials commercially available for FFF include: ABS reinforced with carbon fibers; PLA with carbon fiber, graphite, stainless steel, bronze, brass, copper, bamboo fibers, wood fibers, and iron particles; and PET with carbon fibers. The filler content of these composites is between 5 and 40 vol % [1,24,26,44]. Highly-filled polymeric materials for FFF will be discussed in Section 3 of this review.

The process of ram extrusion of filaments was pioneered by Stratasys and in 1991 they introduced the first AM system of this kind. Their FDM system had two extrusion heads and used two spools of material; one material was used to build the part and the second was used for the support material. Based on the FDM system, a novel system for the manufacturing of multi-material parts was presented by the Rutgers research group, the fused deposition of multiple ceramics (FDMC) [45]. Four extrusion nozzles were included in the system, i.e., four materials could be deposited at the same layer. Different demonstrators, such as piezoelectric components with layers of soft and hard piezoelectric ceramics, were produced. Expiration of the Stratasys patents on the FDM process and growing demand for customized products has driven other companies, such as Beijing Tiertime Technology Co., Ltd., to become emerging competitors in this market [24]. In addition, personal fabrication markets are being encouraged with open source RepRap projects and several small and medium companies are producing FFF machines, such as German RepRap, Aleph Objects, MakerBot Systems, 3D System Inc., Delta Micro Factory Corp., Hage Sondermaschinenbau GmbH & Co KG, EVO-tech, BigRep GmbH, Printbot, Indmatec GmbH, Rokit Inc., Ultimaker, Sharebot srl, MarkForged Inc., 3D Platform, Titan Robotics Ltd., Vixel8, Xery 3D, Prusa Printers, Robox, Zortrax, and Felix printers [24].

#### 2.1.3. Material Extrusion with Screws

The production of rods or filaments represents an additional task that requires special extrusion lines and know-how to obtain filaments or rods with constant cross-sectional area and minimum ovality, which are prerequisites to deposit the adequate amount of material and therefore for a reliable process in fused filament fabrication (FFF) machines. However, not all materials can be made into filaments that can be spooled, but at the same time are rigid enough that they can be pushed by the feeding mechanisms of FFF machines (see Section 2.1.2). Therefore, several research groups and companies are looking into screw-extrusion AM machines that can utilize pellets.

A screw extruder is divided into several zones. In the solid conveying zone pellets are transported to the melting zone, where pellets are softened under heat and friction, and the metering zone in which the molten material is submitted to high pressure before its eviction through the nozzle. The rotating screw has a pumping effect and thus it moves the material from the feeding zone to the nozzle [28]. Controlling the flow of the extruder to deposit the material in a precise manner could be a more challenging task and requires other tools as compared to ram extrusion. Also, the size of the pellets should be controlled in order to obtain a uniform flow of the extruded material [46]. Nevertheless, solutions have been found and below some examples of screw extruder AM machines are described.

Bellini et al. [46,47] developed a system called mini extruder deposition (MED), which consists of a mini screw extruder mounted on three high precision linear motor tables. The three tables were connected to three digital servo drives to monitor the torque, velocity, and rotational speed. The servo drives were also equipped with digital notch filters to eliminate mechanical resonance. The driver’s position, speed, and acceleration of the three axes can simultaneously be controlled. A separate controller was used to regulate the heaters and the motor of the extruding screw. Material temperature was checked at the entrance of the liquefier and closer to the nozzle. Even though the developed preliminary configuration shows opportunities for the use of a wider range of materials, it can only be considered as a starting point for further development, due to the limited information provided by the researchers and the lack of follow-up publications.

Cruz et al. [48] developed their own screw-based extrusion system. The equipment consisted of a vertical single screw extruder with a screw length of 90 mm, a screw diameter of 15 mm, and a die with a diameter of 2 mm. Two band heaters were placed around the barrel to ensure a constant temperature (up to 250 °C) during the plasticization process. The building platform was capable of moving in XYZ directions, controlled by step motors to control the trajectory and the material deposition. The printing process was controlled by a logical controller and a computer was used as an interface to enter the processing conditions (barrel temperature, screw rotational speed, and material rate of deposition) and monitor the process. The designed extruder was capable of processing a feedstock with 59 vol % of carbonyl iron; however, no further details in terms of printability and printed parts were shown.

Two companies have developed screw-based MEAM setups for making small parts and both are currently commercializing their machines. One of them is AIM3D GmbH (Rostock, Germany) [49,50]. The AM machine from AIM3D has two extruders that can take commercially available pellets from thermoplastics or metal injection molding (MIM) feedstock to build a three-dimensional object. The building volume is a cube measuring 255 mm on all sides. As indicated on their website, the only material that is beyond the beta phase of development is a MIM feedstock with stainless steel particles. The second company is Pollen AM Inc. (Paris, France) [51,52]. The Pollen AM MEAM is capable of printing with up to four different materials, and it is also capable of mixing two materials during the printing process. Materials available include unfilled thermoplastics and filled thermoplastic pellets with natural fibers, carbon fibers, minerals, and metal particles [52].

Cincinnati Inc. (Cincinnati, OH, USA) and Oak Ridge National Laboratories (Oak Ridge, TN, USA) have developed a screw extrusion machine for large size additive manufacturing. The setup is called big area additive manufacturing or BAAM. It consists of a single screw extruder mounted vertically on a machine frame, similar to the frames used for laser-based AM machines. The extruder has a feed-rate of 36 kg/h and a unique automatic taping mechanism, which is used to flat the deposited material to increase the contact between deposited layers. The setup is available in two sizes: 7.8 × 3.7 × 3.3 m^3^ and 10.8 × 3.9 × 4.4 m^3^. The motion system is driven by linear motors and the absolute position accuracy is ±0.127 mm. Using BAAM, the manufacturers have been able to print sections of car bodies and sections of buildings. The materials that have been tested include pellets of acrylonitrile-butadiene-styrene (ABS), polyphenylenesulfide (PPS), polyetherketoneketone (PEKK), and polyetherimide (PEI), as well as composites materials containing carbon, glass fibers, and NdFeB particles [53,54].

## 3. Material Extrusion Additive Manufacturing of Highly-Filled Polymers

Highly-filled polymers are compounds of polymers with added particles at concentrations well above 20 vol % in which the interactions between fillers cannot be neglected [55]. In this review we will be talking about compounds with filler contents between 45 and 65 vol %, which can be used for the fabrication of metal or ceramic components. The use of highly-filled polymers for the production of metal or ceramic parts with complex geometries has a long history. Ceramic injection molding (CIM) was the first to be introduced in the 1930s, simultaneously in USA and Germany, for the production of spark plug bodies, but for the next three decades it was of minor interest to the ceramic industry. In the 1960s, CIM was also utilized for the production of ceramic tableware. It was only in the 1970s and 1980s that CIM provided a cost-effective manufacturing method for the mass production of ceramic parts for the automotive industry [56,57]. Metal injection molding (MIM) reached production in the 1970s. In 1979, MIM drew attention when two parts won awards [56]. One part was a screw seal used on a Boeing jetliner. The second part was a niobium alloy thrust chamber and injector for a liquid propellant rocket engine. By the middle 1980s, the MIM sector already had multiple actors [56]. When compared to other manufacturing technologies such as casting and forging, powder injection molding (PIM) is a relatively young technology with great potential.

**Figure 2:**
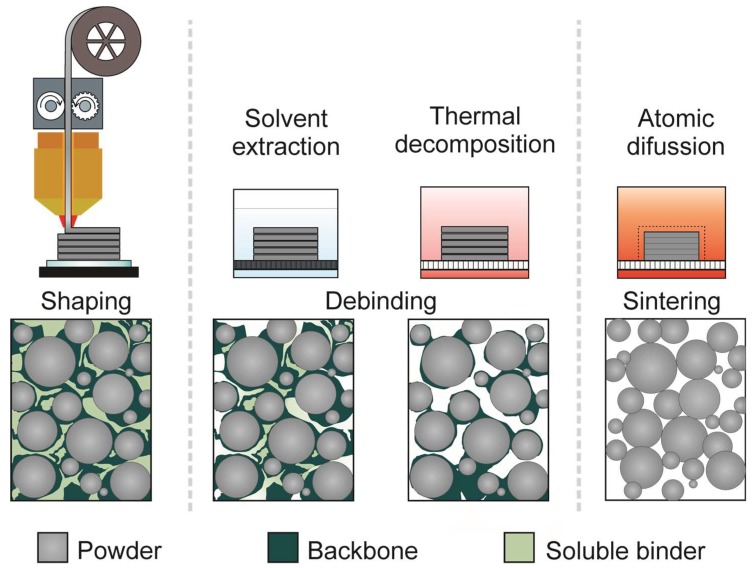
Schematic representation of the shaping, debinding, and sintering (SDS) process and respective morphology of the parts for the fabrication of metal, ceramic, or metal-ceramic components.

The idea of using highly-filled polymers for the additive manufacturing of metal and ceramic parts was first introduced in the 1990s; it was named fused deposition of metals (FDMet) [58,59] and fused deposition of ceramics (FDC) [60,61], respectively. It was based on the Stratasys FDM technology, in which highly-filled polymers with metal or ceramic particles are initially extruded as filaments, and then these filaments are selectively extruded at a temperature higher than the melting point of the binder polymers. Later, as in the case of PIM, the shaping step is followed by the removal of the polymer from the samples using solvents, catalyzers, and/or by thermally decomposition; finally, fully densified metallic or ceramic components are obtained after sintering the parts [58,61,62]. The process is sometimes referred to as shaping, debinding, and sintering (SDS) and a schematic representation of the overall process is shown in Figure 2.

It is important to mention that the SDS process could use other additive manufacturing techniques such as indirect powder bed fusion, binder jetting, vat polymerization, and material jetting. For indirect powder bed fusion, sinterable particles can be coated with a thermoplastic and these coated particles are then fused together with low power lasers, since only the polymer needs to be sintered or melted [63,64]. For binder jetting, sinterable powders can be bound together with resins or adhesives [65,66,67]. As for vat polymerization and material jetting, slurries containing sinterable particles and photopolymerizing resins or thermoplastics can be used to shape parts [68,69,70,71,72]. More details about these processes are given in References [63,64,65,66,67,68,69,70,71,72]. The emphasis of this review is shaping with MEAM, as shown in Figure 2.

MEAM-HP has shown great promise as a cost-effective alternative for the fabrication of metal, ceramic, and metal-ceramic parts [73], particularly for companies currently working with PIM, which already have the equipment and know-how to carry out the subsequent steps of binder removal and sintering to obtain solid parts with complex geometries [73,74]. The production of small metal or ceramic parts by PIM and MEAM-HP are not mutually exclusive; on the contrary, they are complementary. PIM is a technology that becomes economically feasible when large quantities of parts are to be produced (>1000 parts per year), due to the costs associated with the design and manufacture of the mold used in the injection molding machine [57,75]. MEAM-HP is meant to be used for the production of small quantities (i.e., prototypes or custom-made parts) or parts with geometries that cannot be achieved by filling the cavity of a mold. Therefore, we believe that an industrial niche for MEAM-HP will emerge in the near future and thus it is worth investigating and improving the feedstock materials and equipment used in this AM process.

As mentioned before, PIM and MEAM-HP rely on the use of highly-filled polymers; the similarities and differences between their feedstocks will be described in the following section.

### 3.1. PIM and MEAM-HP Feedstocks

Feedstocks for MEAM-HP and PIM are multicomponent systems consisting of a polymeric blend, sinterable powder, and additives. All of these components are needed to fulfil the requirements at different steps of the overall SDS process. Details about these different feedstock components will be described in the following sections.

#### 3.1.1. Binder Systems

The polymeric component of the feedstock is referred to as the binder system. The binder system greatly influences the production process and the quality of the sintered parts, even though it is completely removed during the debinding step. Usually a binder system consists of different types of polymers, waxes, and additives [55]. Generally, three main groups in the binder system can be identified [34,56]:

- The main binder component is the component present in the largest amount and it is removed first during the debinding step. The main binder component represents between 50 and 90 vol % of the total binder system
- The backbone is the component used to hold together the shape of the part while the main binder component is removed during the first debinding stage. The backbone is thermally decomposed prior to sintering. The backbone represents 0 to 50 vol% of the total binder system.
- Additives like dispersant agents, compatibilizers, and stabilizers help to disperse the filler particles in the polymeric binder, preventing agglomeration and phase separation. Additives represent between 0 to 10 vol % of the binder system.

In fact, it is possible to use a binder system with only one polymeric material, but then the debinding step is limited only to thermal degradation, which is a very slow process compared to solvent or catalytic debinding [34,76,77]. For this reason, most binders have at least two components. Table 2, Table 3 and Table 4 show some examples of binder systems components reported in the literature for PIM, MEAM-HP with filaments, and MEAM-HP with pellets or powders, respectively.

As can be seen in Table 2, the main components in the binder system for PIM are polymeric materials with low viscosity (e.g., waxes), materials that dissolve in water (e.g., polyethylene glycol, agar, etc.), or those that undergo catalytic degradation (e.g., polyoxymethylene) [78,79,80]. The majority of backbones are polyolefins (e.g., polyethylene, polypropylene). This is because polyolefins are resistant to many solvents used during debinding, add strength to the debound part, and degrade into hydrocarbons only before sintering. Finally, the most commonly used additive is stearic acid, which facilitates the dispersion of the filler particles. For PIM, low viscosity of the feedstock is required to fill the cavity at lower pressures in the injection molding machine and this is reflected in the binder composition [34].

On the other hand, binder systems for MEAM-HP, which are used as filaments, have components that lead to flexible feedstocks that can be spooled. One way to make feedstocks flexible is to add elastomers [60,61,62,100] or amorphous polyolefins [45]. Another way is to add a stiffer polymer-like polyamide or a polyolefin and add other components that plasticize these polymers to increase their flexibility and at the same time can be dissolved to speed up debinding [101,102]. Other components in the MEAM-HP binders include tackifiers, waxes, and plasticizers. One example of a tackifier used is a hydrocarbon resin, which can improve the adhesion with the previous layers and the flexibility of the filament [45]. Waxes, such as partially crystalline polyolefin wax, can be used to reduce the viscosity and improve the stiffness of filaments [103]. Finally, a low molecular weight polyolefin can be used as a plasticizer to reduce the viscosity of the feedstock [104]. Since finding a formulation that works is a complex task, many times the formulation is not clearly specified in published papers and patents to prevent competitors from using the exact binder systems [58,59,105]. Some formulations available in the literature are shown in Table 3. The binder systems used in MEAM-HP for pellets or powders (Table 4) are a lot more similar to the feedstocks used in PIM; in fact, the goal is to develop machines that can use the readily available PIM feedstocks to 3D print objects. These feedstocks go back to the idea of decreasing the viscosity of the feedstock, thus their main binder components are again waxes or PEG [48,106]. Most of the MEAM-HP methods that do not use filaments are still in the development phase and as such, the feedstock formulations might not be the final formulations that really can be shaped, debound, and sintered.

The effect of binder formulation on the properties of filaments have been investigated and linked to the ability of those filaments to be printed to build a 3D object. For example, Kukla et al. [110] studied the variation in the amount of three different polyolefins as a backbone in the feedstock containing thermoplastic elastomer (TPE, main binder component) and 316L steel powders. It was found that the modulus of the feedstock filaments could be increased with an increase in backbone content. The resulting feedstock properties with medium viscosity (~1000 Pa·s) and medium to high secant modulus (400 to 2700 MPa) were found to be printable in conventional FFF printers in a continuous manner.

Agarwala et al. [118] initially developed a binder system to be used for the production of parts of silicon nitride (Si_3_N_4_) via filaments. The filaments produced with the feedstocks were too brittle to be spooled. However, the high material stiffness enabled the production of parts by hand-feeding the equipment. For this reason, Agarwala et al. [61] later implemented a design of an experiment for optimizing the binder. Four polymers were employed: a backbone polymer, an elastomer for improving the flexibility, a wax for reducing the viscosity, and a tackifier for promoting the adhesion. A tradeoff had to be attained, since the wax reduced the flexibility, and the elastomer increased the viscosity. After the optimization of the system and the pre-treating of the powder with a dispersant for reducing the viscosity, flexible filaments with a diameter of 1.78 ± 0.05 mm could be spooled.

Bhat et al. [121] studied the use of polyethylene systems for the FFF of alumina feedstocks. The designed binders were composed of a polyethylene wax as a plasticizer and a linear low density polyethylene. The plasticizer content varied from 0 to 100 vol % in the binders and in the feedstocks containing 50 vol % of alumina powder. A higher plasticizer content resulted in a lower viscosity, but also in a lower compressive strength of the component. The best properties were attained with feedstocks containing 40 vol % of plasticizer, from which straight filaments could be used for MEAM.

These examples demonstrate that finding the right combination of polymers and their proportions in the binder is not a simple task and for this reason, most of the actual binder formulations that work are not described in detail in the literature. However, it is clear that the selection of the optimal binder systems can be directly based on the compatibility with the powder, resulting viscosity, and mechanical properties (modulus and flexibility) required for MEAM with filaments.

#### 3.1.2. Powder Fillers

Many additive manufacturing technologies rely on the use of powder as their building material; MEAM-HP is not an exception. The powder material used in MEAM-HP is in principle the same metal or ceramic powder as that used in PIM; this represents an advantage compared to AM techniques that rely on powder bed technology, which require a very specific particle size distribution for the process to work properly. In general, MIM utilizes particles with an average size between 5 to 15 μm [34]. Typical mean particle sizes in ceramic CIM are 1 to 2 µm, but also submicron or nano sizes are used in advanced CIM [122]. Therefore, the typical particle sizes used in PIM are fine enough to prevent the plugging of the nozzle (diameter range 0.3 to 0.8 mm) of the MEAM machine.

Many metals and ceramics are available for PIM [34] and in principle they should also work with MEAM-HP; however, not all of them have been tested. Table 5 shows a list of the types of powders that have been successfully shaped by MEAM-HP, debound, and sintered as found in the scientific literature. Table 6 shows the types of materials that are claimed to be available by companies producing MEAM-HP equipment. All companies advertise many other powder fillers as being under development; these materials have been excluded from the list.

Changing the characteristics of the powder can drastically influence the mechanical and flow properties of the feedstock materials, as has been reported in the PIM and the particulate composite literature [127,128,129]. For feedstock materials to be used in FFF this is particularly critical because changing the characteristics of the powder can lead to filaments with low mechanical properties that are not printable.

**Figure 3:**
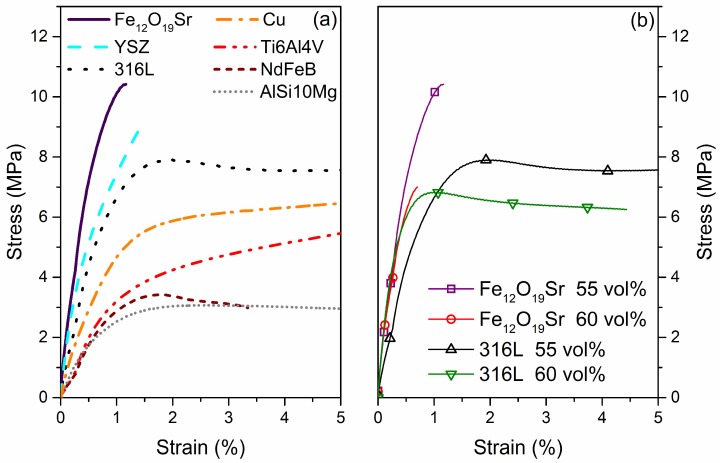
Tensile response of filaments with (**a**) different powders [112] and (**b**) different powder contents [124].

One main factor that affects the properties of highly-filled filaments is the change in the physical and chemical properties of the metal or ceramic fillers. This change can be attributed to the different size, morphology, and chemical composition of the different metals and ceramics, which lead to a distinct interaction with the binder system. Examples of how changing the particles affects the properties of filaments have been presented in the AM literature. For example, Kukla et al. [112] presented the tensile properties of filaments filled with stainless steel (316L), titanium (Ti6Al4V), copper (Cu), rare earth alloy (NdFeB), aluminum (AlSi10Mg), strontium ferrite (Fe_12_O_19_Sr), and yttria stabilized zirconia (YSZ), see Figure 3a. As expected, the tensile properties of the filaments greatly varied as the filler particles were changed and it was observed that the processability of the different filaments varied. Feedstocks containing ceramic fillers (Fe_12_O_19_Sr and YSZ) have the stiffest and less flexible filaments and they have to be fed into the printer head manually, i.e., one strand at a time. Among the metal-filled filaments, 316L was the stiffest, followed by the Cu, Ti6Al4V, NdFeB, and AlSi10Mg. All of the metallic filaments with the exception of NdFeB could be continuously printed on conventional MEAM machines having a feeding unit with counter-rotating wheels. The NdFeB-filled filaments had low stiffness and were fragile and they could only be printed on a MEAM machine with a feeding mechanism consisting of conveyor belts as opposed to rotating wheels. The conveyor belts provided more contact between the feeding mechanism and the filament and prevented the breakage of the filament during feeding into the liquefier unit [112]. It can be concluded that changing fillers will influence the processability of highly-filled filaments and thus different printing conditions or even printing setups might be required to process them by MEAM.

Keeping the same powder type but with different characteristics has also been investigated. For example, Wu et al. [58] studied two 17-4PH stainless steels powders (a spherical powder with an average particle size of 22 µm, and an irregular powder with an average particle size 10 µm). Small powders were preferred for the use of smaller nozzles. In another study, Kukla et al. [111] reported that increasing the average particle size (from 5.5. to 8.6 μm) of round steel particles (316L) used in the filament, while maintaining all parameters constant, can lead to unprintable filaments. The increase in the average particle size resulted in the decrease of apparent viscosity and secant modulus of the filaments by ca. 42% and elongation at break by ca. 35%. The decrease in secant modulus can be linked to a decrease in stiffness and thus it was responsible for the tendency to buckle at the feeding mechanism of conventional FFF machines, resulting in failure during printing. One possible solution to this problem could be to modify the proportion of backbone in the binder system, as discussed previously [111].

The powder content in the filament has also been investigated. Gonzalez-Gutierrez et al. [124] characterized feedstocks and filaments with different contents of Fe_12_O_19_Sr (Figure 5b). It was observed that increasing the content from 55 to 60 vol % made the filaments significantly less ductile (i.e., shorter strain-stress curve); however, the stiffness remained almost the same. The filaments with the highest powder content had to be fed manually and even the printed parts were easily broken if not handled properly during the removal from the building platform. Figure 3b also shows unpublished results from our group in which the 316L steel content in the filaments was increased from 55 to 60 vol %. In the case of steel, the 5 vol % powder increase led to a stiffer filament and, as in the case of Fe_12_O_19_Sr, a more brittle and fragile filament; but contrary to the filaments with Fe_12_O_19_Sr, both steel filaments could be fed continuously from the spool to the printer head. What can be concluded is that the maximum volume content that yields printable filaments is very much material-dependent since the mechanical properties are greatly dependent on the particle-matrix chemical interaction [124].

The processability of filaments by MEAM is not only dependent on the mechanical properties of the filament, but also on the flow properties of the feedstock as well as on the processing conditions, the geometry of the filament, and the design of the printing head. This was discussed by Venkataraman et al. [130] for ceramic and metallic feedstocks with different binder systems. According to these authors, the filament will buckle during the printing process when the extrusion pressure exceeds the critical buckling stress of the material, i.e., $\Delta P^{\prime }>\sigma_{cr}$. The critical stress was considered to be approximately equal to the filament buckling stress by Euler’s criterion. This criterion depends on the geometry of the filament, the elastic modulus (E), and the length between the feeding rollers and the liquefier unit (L). If the filament is a cylinder with radius R, then the critical stress ($\sigma_{cr}$) can be calculated as [131]:

(1) $$\sigma_{cr}\approx \sigma_{E}=\frac{\pi^{2}E}{4{\left(L/R\right)}^{2}}$$

For a non-Newtonian fluid with apparent viscosity $\eta_{a}$, the pressure drop (ΔP) in a capillary rheometer with radius r and length l for a given volumetric rate Q is defined as [132]:

(2) $$\Delta P=\frac{8\eta_{a}Ql}{\pi r^{4}}$$

Venkataraman et al. [130] assumed that there is a linear scaling factor k correlating the pressure recorded in a capillary rheometer with the one during the printing process with the relation $\Delta P=k\Delta P^{\prime }$. Combining this relation with Equations (1) and (2), it can be stated that the filaments will buckle when:

(3) $$E/\eta_{a}<\frac{32Ql{\left(L/R\right)}^{2}}{\pi^{3}r^{4}k}$$

As can be observed in Equation (3), the buckling of the filament will also depend on the radius ($kr^{4}$) and length (l/k) of the nozzle, as well as on the volumetric flow employed (Q/k) during printing. According to the results obtained for feedstocks with different binders and powders, the buckling of the filaments will occur when the $E/\eta_{a}$ parameter is below the experimental critical range of 3 × 10^5^ to 5 × 10^5^ s^−1^ in the range of shear rates commonly employed in filament MEAM (100 to 200 s^−1^) [130].

A similar work was conducted by Rangajaran et al. [133], who investigated the rheology and the mechanical properties of a feedstock containing 55 vol % of Si_3_N_4_. In this case, the parameter k defining the relationship between the pressure measured in the capillary rheometer and the pressure in the FFF nozzle was supposed to be proportional to the diameter difference in both devices and equal to 1.1. Using the same hypotheses as Venkataraman et al. [130], the buckling will occur when $1.1\Delta P>\sigma_{cr}$. The relation was experimentally validated [133], but further research should be conducted dealing with the relation of ΔP with the flow rates, the material properties, and the geometrical parameters of the nozzles.

### 3.2. Effect of Processing on Properties of Feedstocks and Filaments

**Figure 4:**
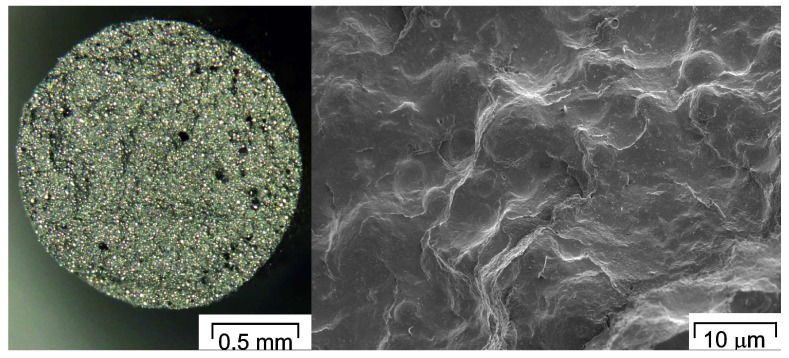
Optical microscopy and SEM images of a filament filled with steel particles [62,73].

Feedstocks for MEAM-HP can be produced in a similar manner as PIM feedstocks: the metal or ceramic powder is mixed with the molten binder constituents and the filler is dispersed in the binder. One of the main requirements is that the resulting compound has a homogeneous distribution of powder particles and binder components. This helps to minimize the segregation of components during the shaping process and later on to obtain isotropic shrinkage after debinding and sintering. Avoiding the segregation of components is crucial to prevent visual defects, excessive porosity, warpage, and cracks in the sintered part [34]. An example of a feedstock material with well-dispersed particles is shown in Figure 4 [62,73]. In the optical micrograph to the left, the shining spots are steel particles and in the SEM micrograph to the right, the particles are fully covered with the binder system, but they are still visible and no agglomerates can be seen.

Feedstocks can be prepared either in batch or continuous processes. Batch operations include roll mill kneaders and mixers, while continuous processing can be done in screw extruders and shear rolls [34]. In general, feedstocks for MEAM-HP have been produced by using a kneader with Z-blades [60,119,120,123], kneader with counter-rotating rollers [62,73], twin roll mill [134], sigma blade mixer [135], and co-rotating twin screw extruder [62,114]. For materials that have a tendency to agglomerate, co-rotating screws or shear rolls might be the best option, since the high shear achieved with the screw design helps to break down agglomerates and disperse the individual particles [136]. Moreover, for hard-to-disperse powders such as Si_3_N_4_, zirconia, and other ceramics, the powder is first coated with surfactants (e.g., oleyl alcohol and stearic acid) during a ball-milling step before blending with the rest of the binder components. After milling, the powder has to be sieved to further remove agglomerates and increase the homogeneity of the fillers in the feedstocks [104,107,133,137]. The temperature of mixing is dependent on the viscosity of the feedstock and the thermal stability of the components used in the binder system. The viscosity of the feedstock determines the shear stress generated during compounding, which in turn determines how well the powder agglomerates are broken and the individual particles are dispersed. Therefore, it is recommended to use higher temperatures for feedstocks with a higher viscosity and lower temperatures for feedstocks with a low viscosity [61].

Filaments are generally produced via extrusion. For example, in capillary rheometers [118,137] for small batches, and in single screw extruders [104,119,120,137] or twin screw extruders [105,137] for larger productions. When dealing with powders prone to agglomeration, extruders with breaker plates and screens can be used to reduce the agglomeration in the filaments; this was observed by Clancy et al. [137] when working with Si_3_N_4_ feedstocks.

It is very important to produce filaments with tight tolerances on the diameter, because the feeding mechanisms in a filament-based MEAM machine sets the feeding speed, and thus the flow rate, based on the assumption that the filament has a constant diameter. If the filament diameter is smaller than the specified value, the flow rate is lower than desirable and thus strands with smaller widths and thicknesses are deposited. This is referred to as underflow and it results in poor bonding between the deposited strands or creates voids between adjacent strands that will not be closed even after sintering. If the diameter is larger than the specified value, the filament cannot be fed into the liquefier or it may lead to overflow. Overflow leads to poor definition of the fine features of the part. Also, the filaments should be as round as possible (i.e., with minimum ovality) to be properly gripped by the feeding mechanism and so ensure constant feed without slippage [61]. In order to produce filaments with constant dimensions and roundness, the extruder is coupled with a conveyor belt or a haul off unit that pulls away the filament, which is finally spooled in a winding unit [114]. Depending on the thermal conductivity of the filament, it may be necessary to cool down the filament by water or by air. Most highly-filled filaments have a high enough thermal conductivity that no water cooling is necessary. The filament’s diameter and roundness need to be monitored with laser micrometers [137] or other optical sensors so that the different processing parameters can be adjusted. Such parameters include, for example, the extruder temperatures and rotational speed, the speed of the conveyor belt, the haul off unit, and the spooling device [114].

The effect of different compounding strategies on the properties of filaments has been observed in zirconia feedstocks [114]. It was observed that the filaments produced after a high shear compounding step in a co-rotating twin screw extruder had much better tensile properties than filaments produced after compounding in a contra-rotating roller mixer. The mechanical properties of both types of filaments are shown in Table 7 [114]. By looking at the values in Table 7, it can be seen that the filament produced after compounding in the twin screw extruder is more ductile and less stiff, since its ultimate tensile strength (UTS) and its elongation at UTS are larger, while its secant modulus is smaller than those of the filament produced after compounding in the roller mixer. Filaments made after twin screw extrusion were easier to print in MEAM machines.

## 4. Building of Parts

The fabrication process is crucial to obtain good quality sintered parts. If there are large gaps between the deposited strands of material and other defects, these defects will remain in the sintered parts, affecting the mechanical performance and functionality of the final part. Therefore, optimizing the printing parameters to obtain a homogeneous part is very important.

The building of parts via MEAM-HP has been reported in the literature. Table 8 summarizes the details of different models of MEAM machines and some parameters used for different types of feedstocks. The setup parameters are highly dependent on the setup design, the binder composition, as well as the type of powder used; therefore, it is clear in Table 8 that all of the parameters are different. Not all of the parameters are described in the literature, so preliminary runs are recommended for new materials in different setups.

### Effect of Processing on Properties of Built Parts

Several studies have been performed to investigate the effect of the printing parameters on the properties of the built parts via MEAM [40,73,138]. It was observed that increasing the temperature of extrusion, bed, and/or envelope improves the adhesion between adjacent strands and as such the mechanical properties. This is due to the increased mobility of the polymeric chains, leading to greater inter-diffusion between the strands [40,138]. In general, decreasing the layer thickness improves the mechanical properties of a built part, but it was found that a certain threshold is needed to avoid over-compression of the deposited strand, which negatively affects the mechanical properties. This threshold is dependent on the material that is being deposited [138]. Finally, the extrusion or volumetric flow rate should be selected carefully depending on the material. For example, Allahverdi et al. [139] conducted the MEAM of advanced electroceramic components using a proprietary binder system with alumina or lead-zirconium-titanate (PZT). It was observed that increasing the volumetric flow rate increased the propensity of the filaments to buckle and therefore the extrusion process was stopped.

Other parameters that affect the quality of the final part are the improperly deposited strands (also referred to as roads) or the spacing between strands, which can lead to incomplete bonding between adjacent strands or between layers. In turn, all of these factors lead to a systematic variation in density and defect appearance in shaped parts [104]. For unfilled polymers or polymers filled with anisotropic fillers, the orientation of the deposited strands and the layering sequence can affect the mechanical properties of the shaped part. During the extrusion and deposition processes, anisotropy can be created due to shear gradients in the nozzle and due to the nozzle movement relative to the building platform. These shear gradients also align the anisotropic fillers in the direction of flow and deposition, and if severe enough, they can result in an orientation-dependent shrinkage and warping during debinding and sintering. Iyer et al. [104] performed systematic printing trials on ceramic-filled feedstocks with printing raster patterns of 0°, 90°, and +45°/−45°. They observed that raster patterns of 0° or 90° led to warping of the built parts, but warpage was avoided when a cross-hatched pattern +45°/−45° was used.

## 5. Post-Shaping Operations

### 5.1. Surface Treatment

**Figure 5:**
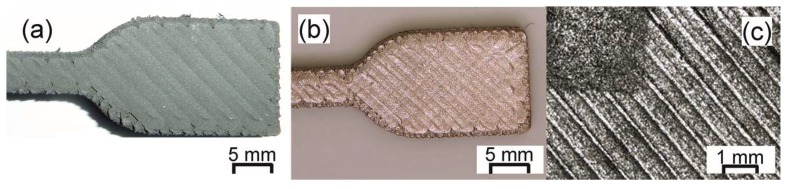
(**a**) 17-4PH steel printed part, (**b**) sintered part without surface treatment, and (**c**) 316L steel sintered part with laser surface treatment in the upper corner and without surface treatment [73,125]. © Carlo Burkhardt (OBE GmbH & Co. KG). Figure 5c first published by EPMA in the World PM2016 Proceedings [125].

After building the part via MEAM, the deposited strands are very noticeable and the lines do not disappear after sintering, as it can be seen in Figure 5a,b for 17-4PH steel parts. When dealing with highly-filled polymers, the strand dimensions cannot be as small as those achieved with unfilled polymers since using very small nozzles results in blockage. The rigid particles inside the feedstock cannot be deformed like the molten binder system and hence they block the nozzle. One possible solution for obtaining a smoother surface is to make thermo-mechanical treatments at the surface. Burkhardt et al. [125] investigated grinding, sandblasting, and laser structuring of shaped parts with 316L steel particles. Grinding and sandblasting for 25 s reduced the depth of the channels at the inter-strand space, but they were not sufficient to fully eliminate the strand marks. Sandblasting also had a negative effect in that it increased any defects in the part related to bad contact between strands (i.e., the spaces between strands were augmented). Finally, the surface treatment with a low power laser for 25 s yielded the best results; the strands were no longer visible and small defects could be covered using this technique (Figure 5c) [125]. The reason for this is that the laser melts the binder system and redistributes it on the surface of the part, carrying with it the filler particles. Treating the surface of green parts can be beneficial when dealing with hard metals or ceramics, which are very difficult to be polished mechanically or will require high energy lasers to alter the surface of the sintered parts.

Another possibility for smoothing the surface of shaped parts could be the use of solvent vapor. Kuo and Mao [140] developed an acetone-vapor system to smooth acrylonitrile butadiene styrene (ABS) parts fabricated by MEAM. A similar technique was also reported by Garg et al. [141] and by Takagishi and Umezu [142]. All groups observed that by partially dissolving the outer surface of the shaped parts the surface roughness was significantly reduced with minimal variations to the geometric accuracy. Similar processes have been described in the informal literature available on the internet, but no literature was found regarding parts fabricated with highly-filled polymers.

### 5.2. Debinding

The polymeric binder system must be removed without disrupting the MEAM-shaped part; this process is commonly referred to as debinding and it is also applicable for parts produced by PIM. Polymers have to be removed completely from the green part since carbon residues can influence the sintering process and affect the quality of the final product in a negative way. Moreover, binder removal is one of the most critical steps in the SDS process [62], since defects can be produced by inadequate debinding, examples of which include bloating, blistering, surface cracking, and large internal voids. There are three main debinding techniques are thermal, solvent, and catalytic methods [34].

The most common way to remove the binder from the shaped/printed parts is to heat the binder so it melts and flows out of the part and/or the binder is thermally degraded and diffuses out of the shaped part. The temperatures used to carry out thermal debinding depend on the formulation of the binders used. For example, Onagoruwa et al. [120] had a binder system consisting of PP/elastomer/tackifier/wax and they heated up their shaped parts in a setter bed of alumina powder. At temperatures lower than 200 °C, some of the binder components melted and they were removed via capillary action, and at temperatures higher than 200 °C, evaporation and thermal decomposition of the residual binders occurred. A similar strategy was employed by Wu et al. [59], but the parts were buried in a carbon powder with a very high surface area to aid the debinding through capillary suction at low temperatures, referred to as wicking. Iyer et al. [104] used a two-stage thermal debinding cycle to remove their binder from the Si_3_N_4_ particles. The first cycle was carried out in a nitrogen atmosphere, where the binder was wicked out of the material using a setter bed. The second cycle was performed in air, where the residual binder was burned off. The composition of the binder system can be modified to improve the thermal debinding rates, which tend to be very slow to prevent damage to the shaped part. This was shown in feedstocks containing 55 vol % piezoelectric powder, in which the proportions of base binder, tackifier, wax, and plasticizer were adjusted not only to improve the processability by MEAM, but also to exhibit good burnout properties to reduce the time required to thermally decompose the binder without any blistering or cracking in the finished samples [45].

**Figure 6:**
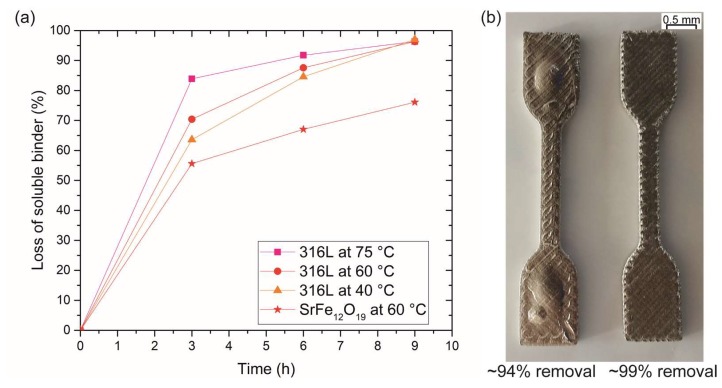
(**a**) Solvent extraction rate for MEAM feedstocks at different temperatures (75, 60, and 40 °C) and different fillers (316L steel and strontium ferrite); (**b**) 17-4PH sintered parts with different levels of solvent extraction [62].

Another way to remove the binder system is to use first a solvent extraction step in a special solvent debinding unit, followed by a thermal debinding step in the same furnace where sintering will take place. This strategy has been investigated in feedstocks consisting of a cyclohexane-soluble thermoplastic elastomer (TPE) and a polyolefin as an insoluble component [112,125]. The solvent extraction rate is a dissolution and diffusion process. Therefore, it is dependent on the temperature, the time, and on the particle characteristics such as the shape and size distribution of the particles. This is illustrated in Figure 6a, where the solvent extraction of feedstocks with 55 vol % 316L steel was performed at different temperatures (75, 60, and 40 °C). After 6 h at 75 °C, 90% of the soluble binder component was removed while only 85% was removed at 40 °C; this is because the dissolution and diffusion of substances increase as the temperature increases. Figure 6a also compares the debinding rate of 55 vol % strontium ferrite-filled feedstocks at 60 °C with that of steel with a similar powder content and temperature. The big difference between the debinding rates of strontium ferrite and steel feedstocks can be attributed to the different particle characteristics. In general, smaller irregular particles (e.g., SrFe_12_O_19_) have more surface area to which the binder system can adhere and smaller pores between the particles; these two factors can slow down the diffusion process first of the solvent and then of the dissolved polymer [112].

When doing solvent debinding, it is important to remove a certain level of the soluble binder component. Gonzalez-Gutierrez et al. [62] showed what happens when the soluble component is not properly removed in Figure 6b. This figure shows two sintered parts, one of them had approximately 94% of the soluble binder removed (left), while the other one had approximately 99% (right). The part with the lower of soluble binder removal had bubbles at the center (left). Bloating of specimens due to incomplete debinding is a common defect in PIM [143]. The cause of this defect is the vapor formed during the degradation of the polymers. In the areas rich in binder, this vapor cannot be easily evacuated and when the partial pressure of the trapped vapor is higher than the atmospheric pressure the bubbles appear [143]. Therefore, the creation of a porous structure by the removal of the soluble component is critical for the following thermal debinding step [62].

**Figure 7:**
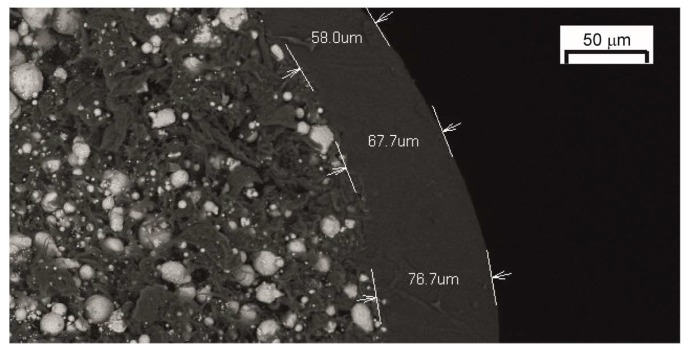
SEM cross-sectional view of POM-based filament produced by BASF SE where a coating layer is observed (courtesy of RHP-Technology GmbH, Seibersdorf, Austria).

The Austrian company EVO-tech GmbH in collaboration with BASF SE offers filaments for MEAM that can be catalytically debound [126]. Such technology is in the process of being patented by BASF SE [116]. The filaments are composed of a polyoxymethylene binder with an external proprietary coating to retain their flexibility. The coating can be observed in the scanning electron microscopy (SEM) image shown in Figure 7. The catalytic debinding process focuses on a solid-to-vapor catalytic degradation of the main binder component. Such catalytic degradation occurs for example when polyoxymethylene-based feedstocks are exposed to acid vapors, such as nitric acid. This results in a much faster binder removal when compared to thermal or solvent debinding [34].

It is worth noting that in solvent and catalytic debinding methods, a skeleton of insoluble or non-degraded polymer remains to impart adequate strength and shape retention up to the onset of sintering. This remaining backbone is thermally removed usually between 200 °C and 600 °C in a pre-sintering step, depending on its chemical composition [34].

### 5.3. Sintering

The last step of the process to obtain dense metal, ceramic, or metal/ceramic parts is sintering. Sintering is a thermal treatment that transforms metallic or ceramic powders into bulk materials. Sintering is performed at temperatures below the melting temperature of the major constituent in the powder, generally within 70 to 90% of the powder’s melting point [34].

Rearrangement, particle movement, and mass transport happen during sintering. Before the sintering occurs, the parts have a highly porous structure formed by particles with a large free surface and therefore a high surface energy. As heat is applied and the temperature increased, the system tends to reduce the surface energy by the formation of solid bonds between the particles. When temperatures increase beyond one half to two-thirds of the melting temperature of the powder material, atomic diffusion and chemical changes on the surface of the particles occur and lead to the formation of solid bonds. These bonds, known as necks [144], continue growing, resulting in the reduction of the porosity and the densification of the part. At the final stage of sintering, the pores are isolated and the density increases up to 99% of the theoretical value [144]. Nevertheless, the increase of the grain growth also occurs in this stage, which hinders the process and the densification rate. At least six different mechanisms for mass transfer involved in sintering have been identified. These mechanisms include: surface diffusion, lattice diffusion, grain boundary diffusion, evaporation-condensation, viscous flow, and plastic flow [144]. All of these mechanisms contribute to the growth of necks between particles and their bonding, leading to an increase in the strength of the consolidated powders. Additionally, some of these mechanisms also lead to the reduction of the porosity and therefore the shrinkage and densification. Surface diffusion is the mechanism that produces surface smoothing, particle joining, and pore rounding, but not densification. If the material has a high vapor pressure, sublimation and vapor transport produce the same effects as surface diffusion. Diffusion along the grain boundaries and through the lattice produces both neck growth and densification. Bulk viscous flow plays an important role in densification, when a wetting liquid is present, while plastic deformation is important when mechanical pressure is applied [34].

**Figure 8:**
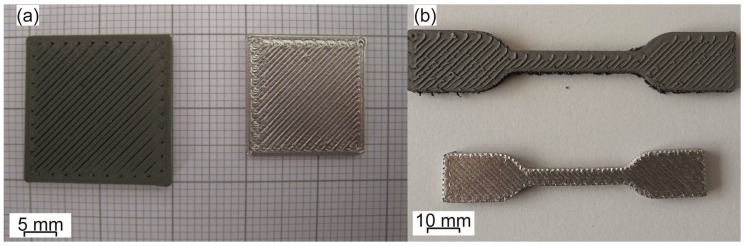
Comparison of printed and sintered parts: (**a**) 316L [125] and (**b**) 17-4PH steels [62].

Shrinkage of the shaped parts by MEAM is always observed after sintering due to the reduction of the porosity and the densification of the parts Examples of printed and sintered parts are shown in Figure 8. Shrinkage and density values obtained in the literature are shown in Table 9.

It is important to mention that the shrinkage does not occur in the same amount in all dimensions, as observed by Lous et al. [145], Agarwala et al. [41], and Kukla et al. [113]. Anisotropic shrinkage has also been reported for parts produced by PIM [146]. The anisotropic shrinkage in PIM is the result of polymer orientation, which can be influenced by the injection molding parameters [147]. Besides polymer orientation, in MEAM-HP, shrinkage and density can be influenced by the presence of gaps between deposited strands. The more gaps, the larger the shrinkage and the lower the density of the sintered parts, since larger gaps cannot be closed during sintering [41]. The shrinkage can also be affected by the orientation of the filler particles, as discussed by Kukla et al. [113]; they observed a smaller shrinkage when the parts were printed on a polypropylene plate, while a larger shrinkage was produced when the parts were printed on top of a permanent magnet, which aligned the anisotropic NdFeB particles in the direction of the magnetic field. Since anisotropic shrinkage is inevitable, it should be included in the CAD design of the parts for the MEAM of ceramics and metals, and the printing strategy needs to be considered and optimized to prevent excessive variation in the shrinkage.

### 5.4. Co-Sintering

One advantage of using MEAM as a shaping technology is that multi-material parts can be easily fabricated compared to powder bed technologies or vat photopolymerization. The multi-material process merely requires a printing head that can work with two or more materials simultaneously. However, the main limitation for applying MEAM-HP for the fabrication of multi-material components is the co-sintering step. For successful co-sintering, the powders have to be sintered together at the same temperature and in the same atmosphere. In order to avoid excessive mechanical stress under cooling, the coefficient of thermal expansion and the shrinkage behavior of both materials should be similar [148].

One of the first demonstrations of the possibility of producing multi-material components was described by Jafari et al. [149]. Filaments with two different piezoelectric ceramics were prepared and parts with alternating layers of these two types of ceramics were produced by MEAM-HP. It was observed that the microstructure and the dielectric properties of the co-sintered parts had no sign of delamination; therefore, it was concluded that MEAM-HP could be used for the fabrication of multi-material transducers with improved performance.

**Figure 9:**
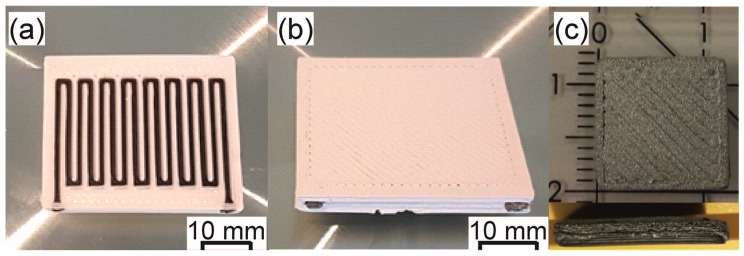
Printed part with a base of zirconia and an insert of 17-4PH steel: (**a**) middle of the printed job, and (**b**) printed job completed. (**c**) Co-sintered part of 17-4PH steel (top) and zirconia (bottom). Please notice that during sintering the zirconia lost its white color due to the reducing atmosphere [114].

More recently, the possibility of producing parts made of 17-4PH steel and zirconia via MEAM-HP has been demonstrated [114,150]. These two powder materials in their commercial state have very different sintering activity since their particle sizes are orders of magnitude different; zirconia has an average particle size around 0.6 μm, while this steel has an average size around 20 μm. In order to increase the sintering activity of the steel powder, it was re-shaped by attrition milling and ball milling. The milling produced particles with a higher specific surface area. Also, after the milling, the initially spherical steel particles became irregular and angularly shaped; thus, the packing of the steel powder was changed and the overall sintering behavior became comparable between zirconia and steel [150]. An example of a part shaped by MEAM with zirconia and steel is shown in Figure 9a,b; a co-sintered part with 17-4PH and zirconia is shown in Figure 9c.

### 5.5. Post-Sintering Operations

Post-sintering operations of the MEAM-printed parts such as the improvement of surface texture, accuracy, aesthetics, and a matte surface finish are often desired depending on the application of the printed part. A simple bead blasting of the surface can help even the surface texture, remove sharp corners from stair-stepping, and give an overall matte appearance. If a smooth or polished finish is desired, then wet or dry sanding and hand-polishing are performed; this is particularly important if no surface treatment was performed before debinding and sintering. In many cases, it is desirable to paint the surface (e.g., with cyanoacrylate or a sealant) prior to sanding or polishing. Painting the surface has the dual benefit of sealing porosity and, by viscous forces, smoothing the stair-step effect, thus making sanding and polishing easier and more effective [2].

Depending on the material used, AM parts can be effectively colored by simply dipping the part into a dye of the appropriate color. If painting is required, the part may need to be sealed prior to painting. Common automotive paints are quite effective in these instances. Another aesthetic enhancement (which also strengthens the part and improves wear resistance) is chrome plating. Ni, Cu, and other coatings can also be applied to the surface for aesthetic improvements. This is particularly important for materials that are sensitive to oxidation and for which the oxidation process causes a significant decay in the wanted properties, for example, the magnetic performance of NdFeB parts [113].

Another post-sintering operation is the infusion process for the production of multi-material parts. The production of metal-ceramic composites was investigated by Onagoruwa et al. [120] and Bandyopadhyay et al. [119]. Onagoruwa et al. [120] developed a PP-based binder which was used for the production of mullite and fused silica preforms with high porosity. The use of MEAM enabled the production of porous ceramic parts in which the pore geometries and connectivity could be controlled. Aluminum was infiltrated into the preforms, obtaining uniform as well as gradient composites. The process was further studied by the authors for the production of aluminum-alumina composites [119]. Fused silica preforms were fabricated by MEAM and sintered. Aluminum was infiltrated into the preforms at ambient atmosphere and the reaction of both components during the infiltration produced ceramic aluminum oxide (with higher stability compared to the fused silica). The displaced silicon moved to the melted metal, and after cooling metal-ceramic composite parts were obtained [119]. Another infiltration process was performed by Bandyopadhyay et al. [151] for the production of lead-zirconium-titanate (PZT)-epoxy composites by two routes. In the direct route, filaments containing 52 vol % of lead-zirconium-titanate (PZT) powder and 48 vol % of proprietary binder were used to produce via MEAM simple shapes with intricate internal structures, such as ladder structures. After debinding and sintering the MEAM-shaped parts, they were infiltrated with an acoustic epoxy resin to form piezocomposites. In the indirect route, molds having the negative structure were created by MEAM using a commercial polymer/wax material. Subsequently, a slurry containing 45 vol % of PZT was infiltrated in the molds and the binders and bonds were burnt out prior to sintering. The sintered parts were then also infiltrated with the acoustic epoxy [151]. Lous et al. [152] used polymer-infiltrated piezoelectric ceramic skeletons fabricated via MEAM-HP to develop transducers for medical imaging; they concluded that the such produced parts had a similar sensitivity to that of a commercial transducer, but the ringing was much longer due to the lack of optimization on the backing layers produced by MEAM-HP.

## 6. Comparison to Other Manufacturing Techniques

MEAM-HP is still in its early stages of development. Therefore, there is not a lot of literature comparing the properties of parts produced by this technique to parts produced by traditional manufacturing methods such as casting and PIM or to other AM techniques such as powder bed fusion, binder jetting, or vat photopolymerization. Just a few examples were found, and they are all presented in this section.

Bandyopadhyay et al. [119] tested the binder developed by Onagoruwa et al. [120] with piezoelectric lead-zirconium-titanate (PZT), comparing production by the direct and indirect methods [119,151]. As previously discussed in Section 5.4, parts were directly produced with filaments of feedstocks containing their binders. However, in the indirect method, molds were first produced using FFF common materials and ceramic slurry was then casted. The electromechanical properties of the thus-fabricated composites were superior to those of the composites solely prepared by casting, due to the control in the phase periodicity obtained in the AM process.

Agarwala et al. [60] prepared silica parts for investment casting with comparable properties to those silica parts produced by conventional core-making techniques by using MEAM. Traditional core-making techniques involve the machining or injection molding of positive patterns of the actual cores and shells in wax or other polymers. These positive molds are then sequentially dipped in a ceramic slurry. The traditional technique is labor-intensive and MEAM, with debinding and sintering, represents a viable alternative. In the same study, Agarwala et al. [60] also compared silica parts shaped by MEAM-HP to parts shaped by PIM. It was observed that the microstructure of sintered parts produced by MEAM-HP had no evidence of delamination or inter-strand debonding, thus the mechanical properties measured were within the acceptable limits of commercial silica parts.

Griffith and McMillin [135] prepared two types of feedstock materials for the production of alumina parts. They mixed a special binder system with the alumina powder and milled the mixture to prepare a powder that could be shaped by the selective laser sintering (SLS) of the binder, debound, and sintered to obtain a full alumina part. They also prepared feedstock materials as discontinuous filaments to shape parts using MEAM. The parts produced by MEAM were debound and sintered under the same conditions as the parts shaped by binder-SLS. After sintering, the density and porosity of the parts were measured. It was observed that the parts shaped by binder-SLS had a much lower density (53 to 65% of theoretical) and higher porosity (36 to 47%) than the parts shaped by MEAM (density 96% of theoretical). The low density obtained by the SLS process indicated that the same feedstock material should not be used for both technologies, but rather an optimized feedstock should be found for each of the technologies. Nevertheless, it was suggested that the parts shaped by binder-SLS could be used to produce ceramic cores for investment casting, while the parts shaped by MEAM could be used in structural applications [135].

## 7. Summary and Future Direction

Material extrusion additive manufacturing (MEAM) is a very popular way to shape three-dimensional objects with thermoplastic materials. However, when using highly-filled polymers (HP) combined with the post-shaping operations of debinding and sintering, metallic, ceramic, and metallic-ceramic parts can be produced.

MEAM can be performed in three main types of setups, which differ in the way the material is fed to the nozzle with either plungers, screws, or as a filament. The use of filaments is the most widespread. In order to carry out the shaping, debinding, and sintering successfully, appropriate feedstock materials have to be developed for each of the MEAM types. The feedstock materials are made by combining different ingredients in a binder system with powder of the desired material. The binder system has to provide enough flowability, when in the molten state, and enough mechanical strength for handling and processing the material before melting and after re-solidifying. The mechanical strength is particularly important when performing filament-based MEAM, since the filaments should not break or buckle during the feeding of the material into the nozzle. The binder system should also thermally decompose or dissolve without affecting the shape of the formed part during the debinding step, and the binder system should degrade and be fully eliminated before sintering of the particles takes place to prevent contamination of the produced part that could lead to poor properties. For this reason, it is clear that the development of such feedstocks is a complicated task and thus the full description of their recipe is not publicly available, since it represents a competitive advantage for their developers.

Once the feedstock material has been developed, the building job has to be optimized by varying the processes parameters and the building strategy (i.e., layer height, temperatures, direction of the deposition, and infill grade), since these factors and other factors will affect the properties and shape of the final part. The debinding of shaped parts by MEAM-HP can be performed in a similar manner as in powder injection molding (PIM), but the majority of results available in the literature are for thermal and solvent debinding. Finally, sintering is conducted the same way as in other powder technologies such as PIM. It is important to point out that there will be a significant anisotropic shrinkage similar to the one observed in powder injection molding; therefore, preliminary trials should be performed to optimize the final shape and performance of parts produced by MEAM-HP, debound, and sintered.

When comparing the results of MEAM-HP with other shaping technologies, it was observed that MEAM-HP produces objects with acceptable properties when the overall process is optimized. After optimization, dense parts of metal, ceramic, or metal-ceramic can be produced for structural applications. However, it is possible that even if the parts retain some porosity after this manufacturing technique, this could be an advantage for certain applications where a large surface area is beneficial; examples of this include filtration, medical applications, or catalytic applications [153,154]. In conclusion, the authors believe that MEAM-HP is a complementary technology to the many other manufacturing technologies available today for the production of metal, ceramic, and multi-material parts, so it is worthwhile to keep improving the materials and processes to make it commercially viable and useful.

Some of the work that could be done in the future to improve MEAM-HP includes:

- The development of improved simulation tools that can predict the properties of built parts by knowing the material properties, processing parameters, and building strategies. This could allow the optimization of the printing process before it is actually done.
- The development of simulations tools for the debinding and sintering processes.
- The development of automatic monitoring systems that supervise the building process that stop and adjust the processing parameters to reduce the amount of unwanted defects on the printed part.
- The development of mechanisms to smooth the surface of the deposited strands as they are being deposited to reduce the surface roughness of the printed parts and to increase the mechanical properties of printed parts.
- The development of new binder systems for filaments and pellets, which are faster to remove and can use other solvents, such as water.
- The development of new feedstock materials with different filler particles that can be co-sintered for the fabrication of new multi-material components with new functionalities.
- The development of simulation tools that could speed up the development of new highly-filled polymers to be used in MEAM. Such simulation tools should take into account the compatibility and interaction of the different polymeric components, as well as the chemical and physical characteristics of powders.
- The improvement of the reliability of screw-based MEAM machines to replace the filaments by pellets, since filaments are hard to make and limit the amount of powder that can be added to the feedstock.
- The improvement of the properties of the interface of multi-material components so as to promote good adhesion between the different materials and obtain multi-material components with a long service life.

As it can be seen, there are areas in research and development in the field of MEAM-HP that should be improved in order to achieve a reliable process that can be used for the fabrication of unique products with multifunctional properties.

## Figures and Tables

**Table 1:** Different additive manufacturing (AM) technologies and descriptions according to EN ISO/ASTM 52921:2017 [11].

AM Process Category	Technologies	Description	Typical Materials	Strengths/Weaknesses
Material extrusion	Fused filament fabrication (FFF) Fused deposition modeling (FDM^TM^) Robocasting	Process in which material is selectively extruded through a nozzle or orifice.	Pellets or filaments of thermoplastic polymers, composites, and highly-filled polymers with metals or ceramics. Highly-filled inks containing a ceramic or metallic powder.	Inexpensive equipment. Great variety of materials. Easy to use.Small to large building spaces. Multi-material parts are possible. / Rougher surface, limited by nozzle radius. Accuracy and speed can be low. Anisotropy of properties. Support structures are needed.
Vat photo-polymerization	Stereolithographic apparatus (SLA^TM^) Digital light processing (DLP^TM^) Scan, spin, and selectively photocure (3SP^TM^) Continuous liquid interface production (CLIP^TM^)	Process which uses photopolymerization. Liquid photopolymer is selectively cured by light (ultraviolet)-activated polymerization.	UV-curable photopolymer resins (with various fillers).	High level of complexity and accuracy. Smooth surface finish. Accommodates large build areas. / Photo-resins only. Liquid monomers can be harmful. Material creeping can occur after curing. Lengthy post-processing needed. Support structures might be needed.Expensive equipment.
Material jetting	Polyjet ^TM^ Smooth curvatures printing (SCP^TM^) Multi-jet modeling (MJM^TM^)	Process in which droplets of build material are selectively deposited.	Photopolymers, thermoplastic polymers, waxes, composites.	High level of accuracy. Allows for full color parts. Multi-material parts are possible. / Support structures are required. Limited number of materials.
Binder jetting	3D printing (3DP^TM^) ExOne VoxelJet	Process in which a liquid adhesive/bonding agent is selectively deposited to join powder materials.	Powdered plastics, metals, ceramics, glass, and sand.	Allows for full color parts. High productivity. Wide range of materials available. / Properties are dependent on the binder used. Post-processing is needed. Powders can be harmful.
Sheet lamination	Laminated object manufacture (LOM) Selective deposition lamination (SDL) Ultrasonic additive manufacturing (UAM)	Process in which sheets of material are bonded to form an object.	Paper, plastic sheets, metal foils/tapes	High volumetric build rates. Relatively low cost (non-metals). Allows for combination of metal foils, including embedding components. / Finishing depends on material used. Post-processing is required. Limited materials. Properties are dependent on the adhesive used.
Powder bed fusion	Selective laser sintering (SLS^TM^) Direct metal laser sintering (SLM^TM^) Electron beam melting (EBM^TM^) Selective heat sintering (SHS^TM^) Multi-jet fusion (MJF^TM^) HP Jet fusion^TM^ High speed sintering	Process in which thermal energy selectively fuses regions of a powder bed.	Plastics, metals, ceramics powders, and sand.	High level of complexity. Powder acts as support material. Wide range of materials available. / Equipment is more expensive. Special powders are required that are more expensive and can be harmful. Powders can age/oxidase fast. Post-processing is generally needed.
Directed energy deposition	Laser metal depositionLaser engineered net shaping (LENS^TM^) Direct metal deposition (DMD^TM^)	Process in which focused thermal energy is used to fuse materials by melting as the material is being deposited.	Metal wire and powders, and powder ceramics	Not limited by direction or axis. Effective for adding features and repairs. Multiple materials can be deposited in a single part. Highest single-point deposition rate. / Powders can be harmful. Finishes depend on material. Post-processing is needed. Limited materials.

**Table 2:** Examples of binder system compositions used in powder injection molding (PIM)**.**

Main Component (50–90 vol %)	Backbone (10–50 vol %)	Additives (1–10 vol %)	Ref.
Carnauba wax	Polypropylene (PP)	Stearic acid	[81]
Paraffin wax	Ethylvinylacetate (EVA)	Stearic acid	[78]
Paraffin wax	High density polyethylene (HDPE)	Stearic acid	[78,81,82,83]
Paraffin wax	Polyethylene (PE), PP	Stearic acid	[84,85,86]
Paraffin wax	HDPE, PP, Polystyrene (PS)	Stearic acid	[87]
Paraffin wax	PE	Stearic acid, oleic acid	[88]
Polyethylene glycol (PEG)	Polymethyl methacrylate (PMMA)	Stearic acid	[89,90,91]
PEG	Polyvinylbutyral (PVB)	Stearic Acid	[92]
PEG	Polyethylene wax	Stearic acid	[93,94]
PEG	Polyimide diisocyanate	2, 6-di-tert-butyl-4-hydroxytoluene	[95]
Polyoxymethylene (POM)	Low density polyethylene (LDPE)	Stearic acid	[77]
POM	Polyolefins	Poly-1,3-dioxepane or poly-1,3-dioxolane or mixtures thereof	[96]
POM	PE	Butanediol formal	[97]
Agar (gel forming polysaccharide)	Glucose	Deionized water, calcium borate, methyl-p-hydroxybenzoate and propyl-p-hydroxybenzoate as biocides	[98]
PEG or polypropylene glycol or polyvinyl alcohol	PS and/or PE	Methylene chloride	[80]
Partially hydrolyzed cold water soluble polyvinyl alcohol	PE or PP	Glycerin, INT-33PA, steric acid, water	[99]

**Table 3:** Examples of binder system compositions used in material extrusion additive manufacturing with highly-filled polymers (MEAM-HP) with filaments.

Main Component (50–90 vol %)	Backbone (0–50 vol %)	Additives (0–10 vol %)	Ref.
Elastomer and wax	Polymer	Plasticizer, tackifier, oleyl alcohol	[60,61,100]
Amorphous polyolefin	Amorphous polyolefin	Tackifier, wax, plasticizer, surfactant	[45,107,108]
Microcrystalline wax	Ethylene Vinyl Acetate (EVA)	None	[103,109]
Thermoplastic elastomer (TPE)	Grafted polyolefin	Unspecified compatibilizer	[62,73,74,110,111,112,113,114,115]
4 hydroxybenzoic acid-behenylester solid, and 4 hydroxybenzoic acid-ethyhexylester	Co-polyamide (PA) 6/12	None	[101]
HDPE	None	Isopropyl tri(dioctyl)pyrophosphato titanate, tri(dioctyl)phosphato zirconate or mixtures thereof	[102]
POM	Polyolefin, and other polymer (polyether, polyurethane, polyepoxide, polyamide, etc)	None	[116]
PA	None	Undisclosed	[105]
Undisclosed	Undisclosed	Stearic acid	[58,59]
LDPE wax	LDPE	None	[117]
Polypropylene	Elastomer	Wax, tackifier, plasticizer	[118,119,120]

**Table 4:** Examples of binder system compositions used in MEAM-HP with pellets or powders.

Main Component (50–100 vol %)	Backbone (0–50 vol %)	Additives (0–10 vol %)	Ref.
PE wax, paraffin wax, PEG	PP	None	[48]
PEG	None	None	[106]
Paraffin wax	LDPE	SA	[117]

**Table 5:** Ceramics and metals investigated for use in MEAM-HP.

Metal or Ceramic	Type	Powder Content in Feedstock (vol %)	Ref.
Ceramic	Silicon nitrate (Si_3_N_4_)	55 and 60	[60,61,104]
Ceramic	Fused silica (SiO_2_)	56, 60, and 65	[60,61,119]
Ceramic	Lead zirconium titanate	50 and 52.6	[60,61,123]
Ceramic	Zirconia	85	[106]
Ceramic	Yttria stabilized zirconia	47	[114]
Ceramic	Strontium ferrite (SrFe_12_O_19_)	53, 55, and 60	[112,124]
Ceramic	Alumina	50	[121]
Ceramic	Mullite + Alumina + MgO	47.93 + 6.85 + 0.69 = 55.47	[120]
Ceramic	Fused silica + MgO	53 + 3 = 56	[120]
Ceramic	Titanium dioxide + MgO	51 + 4 = 55	[120]
Metal	Stainless steel (17-4PH)	55 and 60	[58,59,61,62,73]
Metal	Stainless steel (316L)	50 and 55	[62,74,105,110,111,112,125]
Metal	Stainless steel (AISI 630)	79	[106]
Metal	Tungsten carbide-cobalt	50	[60,61]
Metal	Carbonyl iron	65	[48]
Metal	Titanium (Ti6Al4V)	55	[90]
Metal	Rare earth magnet (NdFeB)	55	[113]

**Table 6:** Feedstocks with powders currently offered by companies; powders in beta phase of development are excluded from the list.

Company	Powders	Ref.
Markforged Inc.	Stainless steels 316L and 17-4PH.	[35]
Desktop Metal Inc.	4140 (chrome moly), copper, Kovar F-15, Inconel 625, 316L (austenitic), 17-4 PH, and tool steel H13	[36]
AIM3D GmbH	Stainless steels 17-4PH, 316L, 410L, 430 and 440C, tool steel M2, and low-alloy steel 4340	[50]
EVO-tech GmbH	Stainless steel 316L	[126]

**Table 7:** Tensile properties of filaments produced by different compounding strategies [114].

Compounding Method	Ultimate Tensile Strength—UTS (MPa)	Elongation at UTS (%)	Secant Modulus (MPa)
Twin screw extruder	13.7	3.07	1250
Roller mixer	10.6	1.28	1860

**Table 8:** Equipment and processing parameters used to process materials for MEAM-HP.

MEAM Model	Fillers in Feedstocks	Building Parameters Given	Refs.
Stratasys FDM^TM^ 1650 (Filament-based)	Mullite, fused silica, and titanium dioxide	Ext. Temp: 235–237 °C Envelope Temp: 48 °C Material flow rate: 130%	[120]
Hage3D-72L (Filament-based)	Stainless steel 316L at 55 vol %	Nozzle diameter: 0.5 mm Ext. Temp: 240 °C Deposition speed: 50 mm/s	[125]
Hage3D-72L (Filament-based)	Yttria stabilized zirconia at 47 vol % Modified stainless steel 17-4PH powder at 47 vol %	Ext. Temp: 220–240 °C Bed Temp: 20 °C Print speed: 10 mm/s Layer thickness: 0.25 mm	[114]
Wanhao Duplicator i3 v2 (Filament-based)	Stainless steel 316L and 17-4PH at 55 vol %	Printing surface: glass or PP Nozzle diameter: 0.6 mm Ext. Temp: 210–260 °C Bed Temp: 60 °C Flow rate: 100–200% Deposition speed: 40–80 mm/s Layer thickness: 0.15–0.20 mm	[62,73]
Ultimaker 2 (Filament-based)	Stainless steel 316L	Nozzle diameter: 0.8 mm Feed speeds: 0.5–7 mm/s Deposition speed: 14 mm/s Ext. Temp: 235–240 °C Built rate: 0.62–5 mm^3^/s	[105]
Stratasys 3D Modeler (Filament based)	Si_3_N_4_ (Honewell’s GS44 grade)	Nozzle diameter: 0.25 mm Ext. Temp: 185 °C Envelope Temp: 37 °C Layer thickness: 0.254 mm	[104]
Mini-Extruder Deposition (MED) (Screw-based)	Piezoelectric ceramic ECG9/PZT [107]	Top liquefier temp.: 145–160 °C Lower liquefier temp.: 135–145 °C Nozzle diameter: 0.3 and 0.6 mm Pellet size: 1–5 mm	[46]
Fused Deposition of Metals (FDMe)(Screw-based)	Carbonyl iron at 57 to 59 vol %	Top liquefier temp.: 155–159 °C Lower liquefier temp.: 180–18 7 °C Nozzle diameter: 2 mm	[48]

**Table 9:** Linear shrinkage and density after the sintering of parts produced by MEAM-HP.

Material	Linear Shrinkage (%)	Percentage Density from Theoretical (%)	Ref.
Fused silica	1–4	70	[60]
Mullite	10–12	N/A	[120]
Fused silica	8–12	N/A	[120]
316L stainless steel	19.2 ± 0.02	95	[125]
Piezoelectric ceramics	16–20	N/A	[145]
Silicon nitride	12–20	95–98	[41]
NdFeB	16–19	94–96	[113]
